# Fucoidan Alleviates Acetaminophen-Induced Hepatotoxicity via Oxidative Stress Inhibition and Nrf2 Translocation

**DOI:** 10.3390/ijms19124050

**Published:** 2018-12-14

**Authors:** Yu-qin Wang, Jin-ge Wei, Meng-jue Tu, Jian-guo Gu, Wei Zhang

**Affiliations:** 1 Department of Pharmacology, School of Pharmacy and Key Laboratory of Inflammation and Molecular Drug Target of Jiangsu Province, Nantong University, Nantong 226001, China; weijinge1@sina.com (J.W.); dreamjojojo@sina.com(M.T.); jgu@tohoku-mpu.ac.jp (J.G.); 2 Division of Regulatory Glycobiology, Institute of Molecular Biomembrane and Glycobiology, Tohoku Medical and Pharmaceutical University, Sendai, Miyagi 981-8558, Japan

**Keywords:** fucoidan, acetaminophen, Nrf2, oxidative stress, hepatotoxicity

## Abstract

Acetaminophen (APAP) is a widely used analgesic and antipyretic drug that leads to severe hepatotoxicity at excessive doses. Fucoidan, a sulfated polysaccharide derived from brown seaweeds, possesses a wide range of pharmacological properties. However, the impacts of fucoidan on APAP-induced liver injury have not been sufficiently addressed. In the present study, male Institute of Cancer Research (ICR) mice aged 6 weeks were subjected to a single APAP (500 mg/kg) intraperitoneal injection after 7 days of fucoidan (100 or 200 mg/kg/day) or bicyclol intragastric administration. The mice continued to be administered fucoidan or bicyclol once per day, and were sacrificed at an indicated time. The indexes evaluated included liver pathological changes, levels of alanine aminotransferase (ALT) and aspartate aminotransferase (AST) in the serum, levels of reactive oxygen species (ROS), malondialdehyde (MDA), superoxide dismutase (SOD), glutathione (GSH) and catalase (CAT) in the liver, and related proteins levels (CYP2E1, pJNK and Bax). Furthermore, human hepatocyte HL-7702 cell line was used to elucidate the potential molecular mechanism of fucoidan. The mitochondrial membrane potential (MMP) and nuclear factor-erythroid 2-related factor (Nrf2) translocation in HL-7702 cells were determined. The results showed that fucoidan pretreatment reduced the levels of ALT, AST, ROS, and MDA, while it enhanced the levels of GSH, SOD, and CAT activities. Additionally, oxidative stress-induced phosphorylated c-Jun N-terminal protein kinase (JNK) and decreased MMP were attenuated by fucoidan. Although the nuclear Nrf2 was induced after APAP incubation, fucoidan further enhanced Nrf2 in cell nuclei and total expression of Nrf2. These results indicated that fucoidan ameliorated APAP hepatotoxicity, and the mechanism might be related to Nrf2-mediated oxidative stress.

## 1. Introduction

Drug-related hepatotoxicity is a common adverse event in clinic, since a large number of drugs are metabolized in the liver [1,2]. Acetaminophen (APAP) is a widely used analgesic and antipyretic drug, which is safe at therapeutic doses. However, severe hepatotoxicity resulting from excessive doses is the leading cause of acute liver failure in the western world [3]. At therapeutic doses, APAP is mainly metabolized to nontoxic water-soluble metabolites by glucuronidation and sulfation in the liver, and only a small fraction of APAP is metabolized by various cytochrome P450s (CYPs) such as CYP2E1, CYP3A4, CYP1A2, and CYP1A1 to a toxic active product, *N*-acetyl-p-benzoquinoneimine (NAPQI) [4,5]. NAPQI forms APAP-glutathione, APAP-cysteine, and APAP-*N*-acetylcysteine by conjugating with glutathione (GSH) [6]. However, an APAP overdose exhausts glucuronidation and sulfation pathways and produces excess NAPQI, subsequently consuming GSH excessively. Excessive NAPQI forms cysteine adducts by binding to hepatocyte proteins, triggering oxidative stress, mitochondrial dysfunction, cellular necrosis, apoptosis, and even hepatic injury [7].

Nuclear factor-erythroid 2-related factor 2 (Nrf2), a crucial transcription factor, is required for the progress of various diseases, particularly those arising from oxidative stress [8]. Upon exposure to stressors or inducers, Nrf2 enters the nucleus from the cytoplasm and binds to antioxidant response elements (ARE), then activates downstream target genes, including nicotinamide adenine dinucleotide phosphate (NAD(P)H)-quinone oxidoreductase 1 (NQO1) and heme oxygenase-1 (HO-1). It has been reported that Nrf2-deficient mice have a greater severity than wild-type mice in APAP-induced liver injury [9]. In addition, activation of Nrf2/ARE signaling alleviates cerebral ischemia and reperfusion injury by inhibiting reactive oxygen species (ROS) generation and neuronal apoptosis [10]. Thus, targeting Nrf2 activation might be effective for the prevention of hepatotoxicity. Furthermore, oxidative stress also contributes to the phosphorylation of c-Jun N-terminal protein kinases (JNKs) [11]. The translocation of phosphorylated JNK to the mitochondria further aggravates the mitochondrial oxidant stress and evokes mitochondrial dysfunction and DNA fragmentation, thus ultimately causing hepatocyte necrosis [12]. 

Currently, in addition to *N*-acetylcysteine (NAC), there is no other effective drug for the treatment of liver damage caused by excess APAP. However, it is most beneficial only at an early phase of APAP intoxication, and the efficacy decreases at later times [13]. Therefore, it is urgent to explore novel candidates in preventing and treating APAP-induced hepatotoxicity. Fucoidan, which is mainly found in various species of brown seaweed, is a sulfated polysaccharide containing l-fucose and sulfate groups. Numerous studies have indicated that fucoidan exhibited several therapeutic properties both in vivo and in vitro, such as anticoagulant, antitumor, antiviral, antidiabetic, and anti-inflammatory activities [14]. It has been reported that cotreatment of fucoidan ameliorated APAP-induced liver damage and cell death in rats [15]: However, the impacts of fucoidan on APAP-induced liver injury have not been sufficiently addressed yet. Bicyclol, a synthetic antihepatitis drug in China, possesses protective effects against experimental liver injury induced by several chemical toxins and alcohol [16]. Since the hepatoprotective mechanism of bicyclol is partly related to the clearance of ROS [17], it was used as the positive control in this study. The aim of the present study was to investigate the effect of fucoidan on APAP-induced liver injury and to explore potential molecular mechanisms. 

## 2. Results 

### 2.1. Fucoidan Attenuated APAP-Induced Acute Liver Injury in Mice

**Figure 1:**
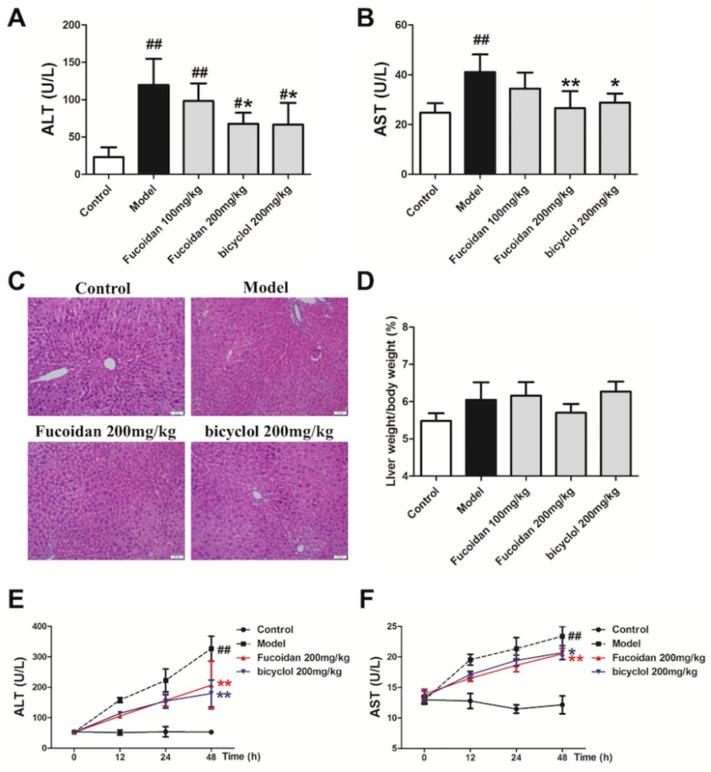
Fucoidan attenuated acetaminophen (APAP)-induced acute liver injury in mice. Male Institute of Cancer Research (ICR) mice were randomly divided into control, model, fucoidan 100 mg/kg, fucoidan 200 mg/kg, and bicyclol 200 mg/kg groups. After pretreating with fucoidan or bicyclol for 7 days, the acute hepatic injury model was induced by intraperitoneal injection of 500 mg/kg APAP, and the normal group was injected with an equal amount of saline. The mice continued to be administered fucoidan or bicyclol once per day, and were sacrificed at an indicated time. (**A**) Serum level of aminotransferase (ALT) in different groups 4 h after intraperitoneal injection of APAP (*n* = 8). (**B**) Serum level of aspartate aminotransferase (AST) in different groups 4 h after intraperitoneal injection of APAP (*n* = 8). (**C**) Representative images of hematoxylin and eosin (H&E) stained liver sections from different groups after APAP treatment (bar = 50 μm). (**D**) Liver ratio in different groups after APAP treatment (*n* = 8). (**E**) Serum levels of ALT at different time points after APAP injection (*n* = 6). (**F**) Serum levels of AST at different time points after APAP injection (*n* = 6). Data are expressed as mean ± SD, *^#^ p* < 0.05, *^##^ p* < 0.01 versus control group; * p < 0.05, ** p < 0.01 versus model group.

The serum levels of alanine aminotransferase (ALT) and aspartate aminotransferase (AST) are usually used as biomarkers for evaluating hepatic function [18,19]. To explore the role of fucoidan against APAP hepatotoxicity, first we detected serum ALT and AST levels in different groups. Starting from 4 h after APAP injection, a massive hepatic toxicity was induced, as revealed by increased serum levels of ALT and AST (Figure 1A,B), and these elevations continued until 48 h after APAP administration (Figure 1E,F). These results indicated that the APAP-induced acute liver injury model was successful, whereas the increased serum levels of ALT and AST were significantly inhibited in the mice treated with fucoidan and bicyclol. Furthermore, the histological examination corroborated the serum ALT and AST results. As shown in Figure 1C, APAP-induced accumulation of erythrocytes in the central vein and sinusoids, infiltration of inflammatory cells, and disordered arrangement in hepatocytes were effectively attenuated by fucoidan or bicyclol treatment. However, the liver ratio in different groups had no significant difference after APAP administration (Figure 1D). These results indicated that treatment with fucoidan or bicyclol prevented APAP-induced acute liver injury. 

### 2.2. Fucoidan Enhanced Antioxidant Capacity and Reduced the Levels of Malondialdehyde (MDA) and ROS in the Liver of APAP-Treated Mice

**Figure 2:**
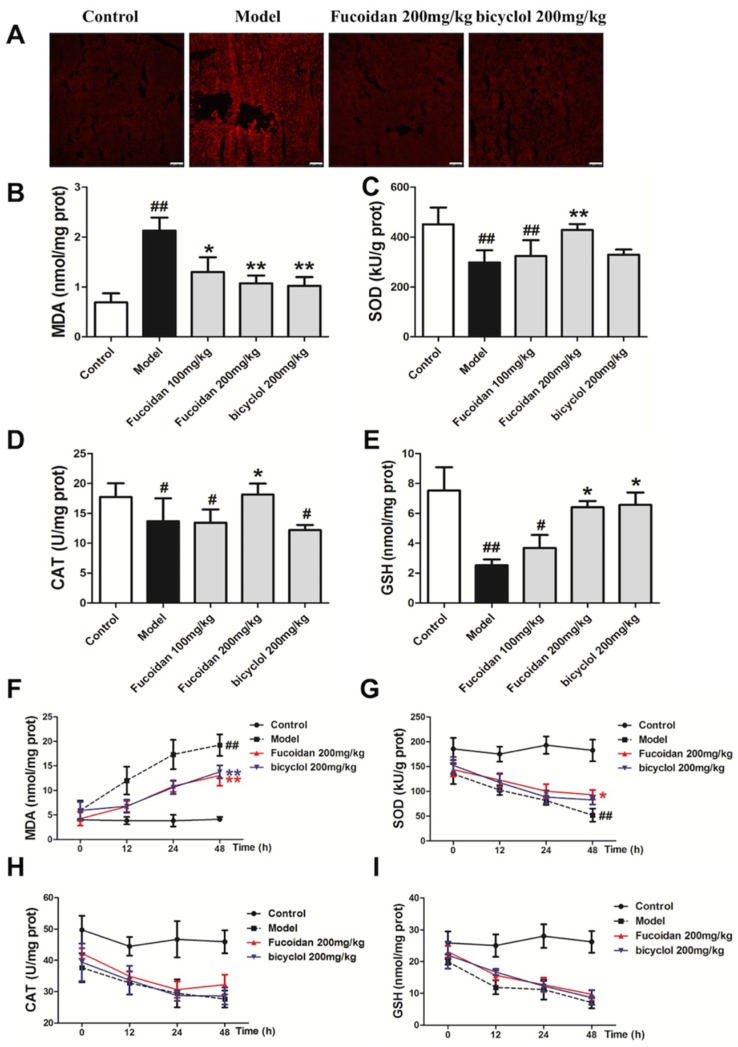
Fucoidan inhibited APAP-induced oxidative stress in mice. Male ICR mice were randomly divided into control, model, fucoidan 100 mg/kg, fucoidan 200 mg/kg, and bicyclol 200 mg/kg groups. After pretreating with fucoidan or bicyclol for 7 days, the acute hepatic injury model was induced by intraperitoneal injection of 500 mg/kg APAP, and the normal group was injected with an equal amount of saline. The mice continued to be administered fucoidan or bicyclol once a day, and were sacrificed at an indicated time. Hepatic tissues were taken for frozen sections and other detections. (**A**) Hepatic sections (4 h) were stained with dihydroethidium (DHE) fluorescent dye, and the levels of superoxide anion were observed with a confocal microscope (bar = 100 µm). (**B**,**F**) Malondialdehyde (MDA) and (**E**,**I**) glutathione (GSH) levels, (**C**,**G**) superoxide dismutase (SOD) and (**D**,**H**) catalase (CAT) activities in the liver were measured for evaluating the level of hepatic oxidative stress. Data are expressed as mean ± SD, *n* = 8, *^#^ p* < 0.05, *^##^ p* < 0.01 versus control group; * p < 0.05, ** p < 0.01 versus model group.

Overdose of APAP produces excessive NAPQI, which binds to hepatocyte proteins to form cysteine adducts, leading to cellular processes such as oxidative stress, mitochondrial damage, and ultimately hepatocytes necrosis. Oxidative stress is critical in the pathophysiological mechanism of hepatic injury [12,20], and ROS may be the common pathogenesis of hepatic disease [21,22]. ROS fluorescence probe–dihydroethidium (DHE) staining was performed on frozen sections of mice liver. As shown in Figure 2A, fluorescence intensity in the model group significantly increased at 4 h after APAP exposure, and meanwhile, pretreatment with fucoidan and bicyclol attenuated the increase of fluorescence intensity. The level of MDA suggested tissue lipid peroxidation, and the response was consistent with the ROS staining (Figure 2B,F). APAP-induced oxidative stress is normally detoxified by the enzymatic antioxidant defense system. At 4 h after APAP exposure, GSH level, superoxide dismutase (SOD), and catalase (CAT) activities remarkably decreased compared to those of the control group, whereas treatment with fucoidan or bicyclol attenuated the above changes (Figure 2C–E). With the aggravation of liver damage, fucoidan continued its protective effects against hepatotoxicity by enhancing SOD activity at 48 h after APAP injection (Figure 2G). However, no significant change in CAT activity and GSH content was observed at other time points after APAP administration (Figure 2H–I). Thus, we speculated that fucoidan postponed the consumption of GSH in the early phase of liver injury, and this effect was significantly diminished in the later stage. These data suggested that fucoidan treatment might reduce oxidative stress and restore endogenous antioxidant systems to prevent APAP-induced hepatotoxicity. 

### 2.3. Fucoidan Decreased Phosphorylation of JNK and Expression of Bax in the Liver of APAP-Treated Mice

**Figure 3:**
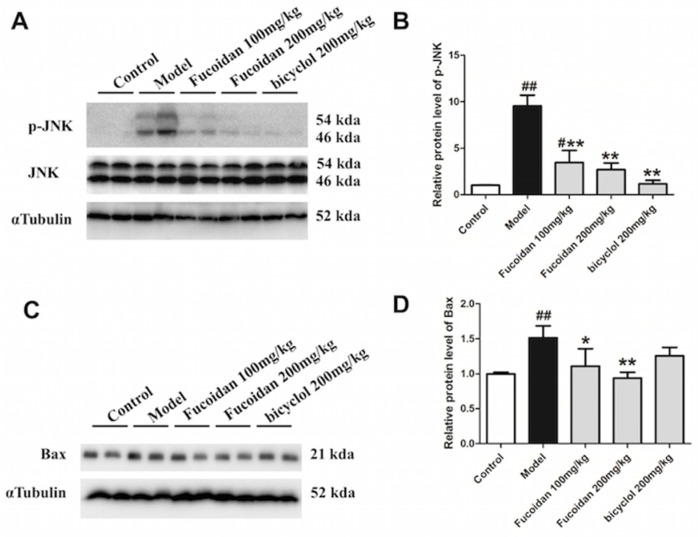
Fucoidan inhibited APAP-induced c-Jun N-terminal protein kinase (JNK) phosphorylation and Bax protein expression in mice. Male ICR mice were randomly divided into control, model, fucoidan 100 mg/kg, fucoidan 200 mg/kg, and bicyclol 200 mg/kg groups. After pretreating with fucoidan or bicyclol for 7 days, the acute hepatic injury model was induced by intraperitoneal injection of 500 mg/kg APAP, and the normal group was injected with an equal amount of saline. Four hours after injection, the hepatic tissue was taken for further detections. The (**A**,**B**) phosphorylation level of JNK and (**C**,**D**) protein expression of Bax were determined by Western blotting analysis. The quantification of relative protein expression was performed by densitometric analysis, and α Tubulin was used as an internal control. All results were expressed as mean ± SD, *n* = 6, *^#^ p* < 0.05, *^##^ p* < 0.01 versus control group; * p < 0.05, ** p < 0.01 versus model group.

It is well known that excessive ROS induced by APAP results in phosphorylation of JNK, which further amplifies mitochondria oxidative stress [23]. Moreover, the pro-apoptotic protein Bax, which belongs to the Bcl-2 family of proteins, is highly expressed exposed to an APAP overdose [24,25,26]. The pathophysiological importance of Bax in APAP hepatotoxicity has been shown by the temporary inhibition of nuclear DNA fragmentation and delayed cell death in Bax-deficient mice [27]. In the present study, the APAP-induced upregulation of phosphorylated JNK was abrogated by pretreatment with fucoidan and bicyclol (Figure 3A,B). Additionally, fucoidan and bicyclol also attenuated the increased protein expression of Bax after APAP injection (Figure 3C,D). Our results indicated that fucoidan, like bicyclol, significantly suppressed JNK phosphorylation and Bax protein expression, and then protected hepatocytes from APAP-induced damage.

### 2.4. Fucoidan Attenuated APAP-Induced Acute Injury in HL-7702 Cells

**Figure 4:**
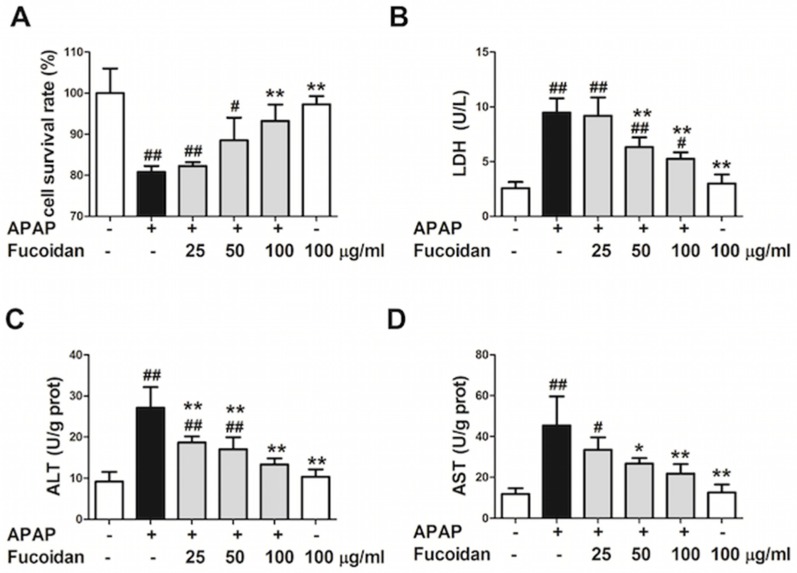
Effect of fucoidan on APAP-induced acute injury in HL-7702 cells. Cells were treated with various concentrations of fucoidan (25, 50, or 100 μg/mL) for 4 h and subsequently stimulated with APAP (20 mM) for 24 h. (**A**) MTT assay was used to observe the survival rate of HL-7702 cells. (**B**) The levels of lactate dehydrogenase (LDH), (**C**) ALT, and (**D**) AST were determined to evaluate the damage of cells. Similar results were obtained from three independent experiments. All results were expressed as mean ± SD, *n* = 3, *^#^ p* < 0.05, *^##^ p* < 0.01 versus control group; * p < 0.05, ** p < 0.01 versus APAP-treated group.

Previous results have indicated that pretreatment with fucoidan attenuated APAP-induced acute liver injury in vivo. In addition, many studies have reported that fucoidan exhibited several biological activities in vitro [14]. In order to elucidate the protective mechanism of fucoidan on APAP-induced liver injury, an acute APAP injury model was established in human normal hepatocyte HL-7702 cell line. 3-[4,5-dimethylthiazol-2-yl]-2,5- diphenyl tetrazolium bromide (MTT) assay was used to observe the effect of fucoidan pretreatment on the survival rate of HL-7702 cells injured by APAP. As shown in Figure 4A, the survival rate of cells was significantly inhibited by incubation with 20 mM APAP for 24 hours, while pretreatment with 50 or 100 µg/mL fucoidan significantly rescued the decreased cell survival rate. The result was further confirmed by detection of lactate dehydrogenase (LDH) in the culture medium (Figure 4B). In addition, levels of ALT and AST were markedly increased due to incubation with APAP, while fucoidan pretreatment ameliorated the damage of APAP to HL-7702 cells (Figure 4C,D).

### 2.5. Fucoidan Enhanced Antioxidant Capacity and Reduced the Levels of MDA and ROS in APAP-Damaged HL-7702 Cells

**Figure 5:**
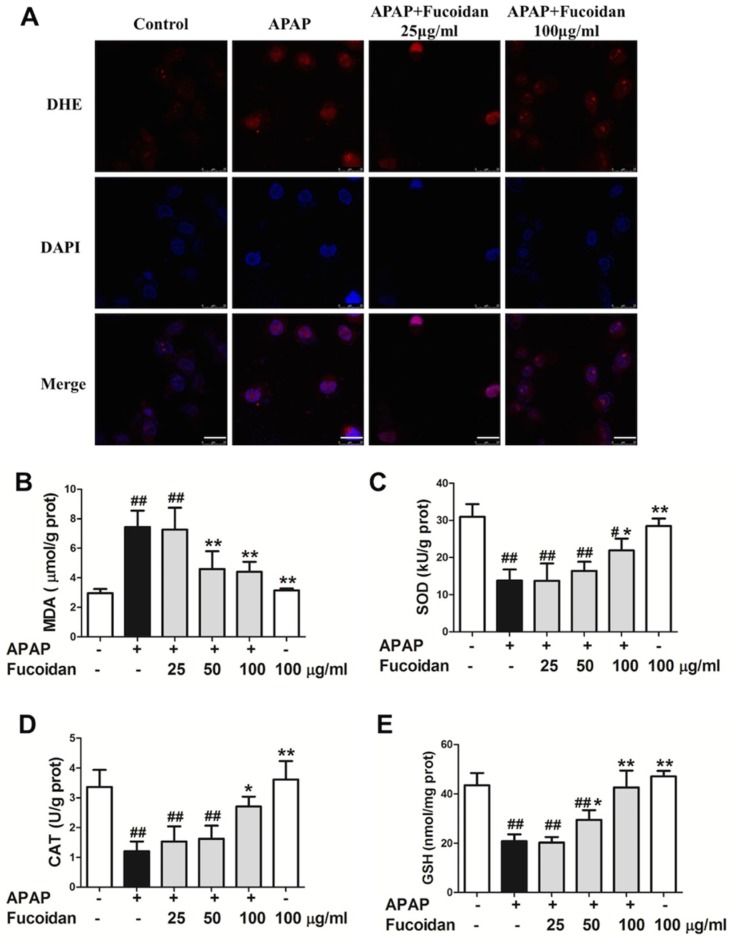
Effect of fucoidan on APAP-induced oxidative stress in HL-7702 cells. Cells were treated with various concentrations of fucoidan (25, 50, or 100 μg/mL) for 4 h and subsequently stimulated with APAP (20 mM) for 24 h. (**A**) HL-7702 cells were stained with DHE fluorescent dye (red), and the levels of reactive oxygen species (ROS) were observed with a confocal microscope (Bar = 25 µm). The nuclei were stained with DAPI (blue). The levels of (**B**) MDA, (**C**) SOD, (**D**) CAT, and (**E**) GSH were measured to evaluate the level of oxidative stress in cells. Similar results were obtained from three independent experiments. All results were expressed as mean ± SD, *n* = 3, *^#^ p* < 0.05, *^##^ p* < 0.01 versus control group; * p < 0.05, ** p < 0.01 versus APAP-treated group.

As shown in Figure 5A, DHE staining showed that the fluorescence intensity was remarkably enhanced in APAP-treated cells. Comparing to the model group, fucoidan attenuated the fluorescence intensity of DHE staining after APAP incubation. Moreover, the variation trend of MDA was similar to that of ROS (Figure 5B). Furthermore, compared to the control group, an overdose of APAP resulted in a significant decrease in GSH, CAT, and SOD activities after 24 h of APAP administration. However, as expected, pretreatment with fucoidan (100 µg/mL) induced a significant increase in GSH content, CAT, and SOD activities (Figure 5C–E). These data suggested that fucoidan attenuated APAP-induced oxidative stress by upregulating the activities of antioxidant enzymes in HL-7702 cells.

### 2.6. Fucoidan Suppressed the Protein Expression of CYP2E1 Both In Vivo and In Vitro

**Figure 6:**
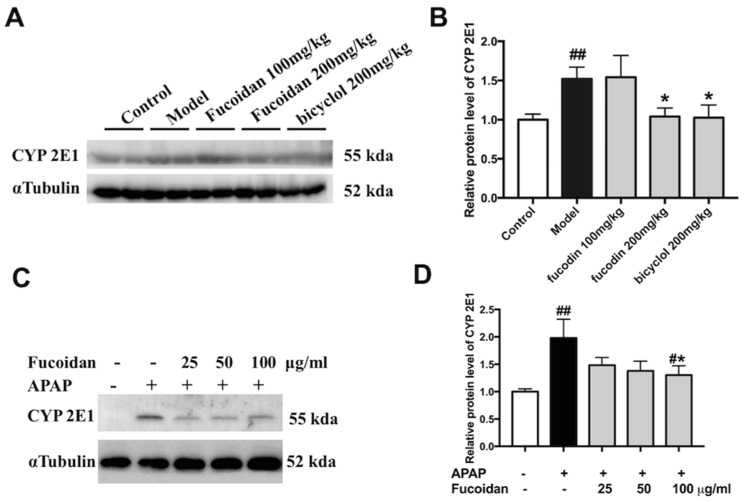
Effect of fucoidan on protein expression of CYP2E1 following an APAP overdose. (**A**) Representative blots of CYP2E1 and α Tubulin expression in hepatic tissues. (**B**) Quantification of CYP2E1 protein levels in liver tissues (*n* = 6). (**C**) Representative blots of CYP2E1 and α Tubulin expression in HL-7702 cells. (**D**) Quantification of CYP2E1 protein levels in HL-7702 cells (*n* = 3). Similar results were obtained from three independent experiments. All results were expressed as mean ± SD, *^##^ p* < 0.01 versus control group; * *p* < 0.05 versus APAP-treated group.

It is widely accepted that the highly reactive intermediate of NAPQI is metabolized by the CYP pathway (primarily by CYP2E1) [4]. According to the data of protein expression levels of CYP2E1 using Western blot, we observed that APAP alone enhanced the protein levels of CYP2E1 both in liver tissues and in HL-7702 cells. Meanwhile, with fucoidan or bicyclol, the expression of CYP2E1 was significantly suppressed (Figure 6). These findings suggested that the hepatoprotective effect of fucoidan might be partially associated with the suppression of CYP enzymes.

### 2.7. Fucoidan Attenuated APAP-Induced Alteration of Mitochondrial Membrane Potential and Phosphorylation of JNK in HL-7702 Cells

**Figure 7:**
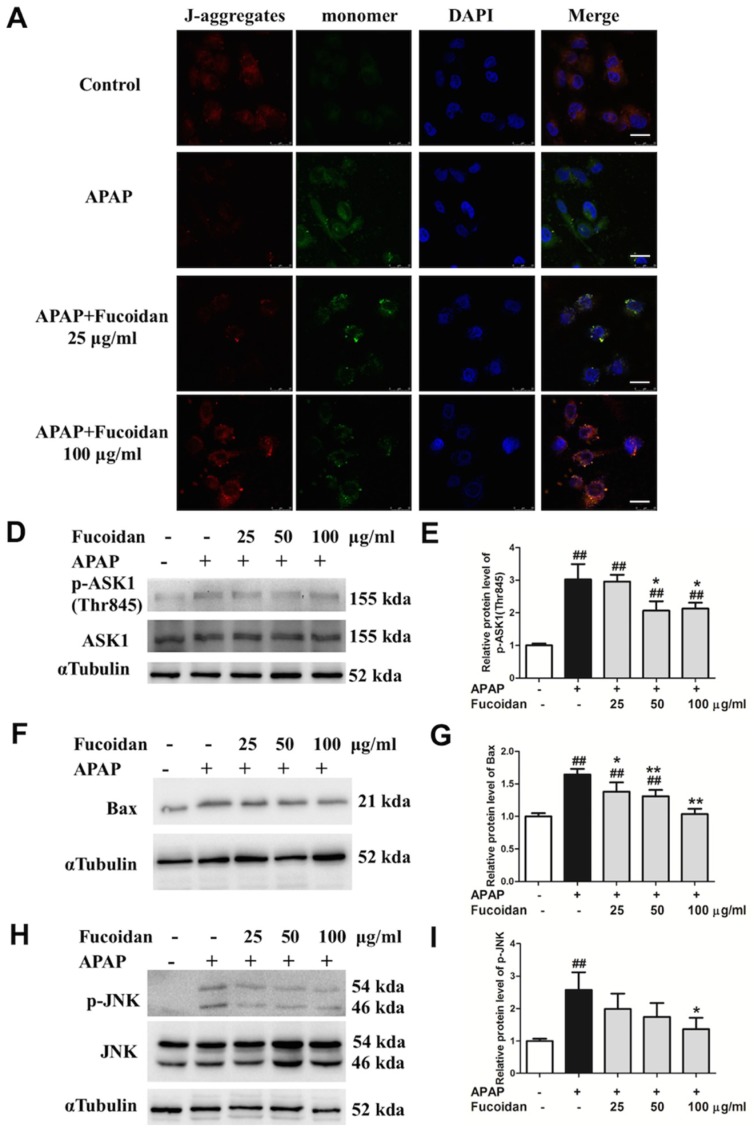
Effect of fucoidan treatment on APAP-induced mitochondrial membrane potential (MMP) and the JNK pathway in HL-7702 cells. Cells were treated with various concentrations of fucoidan (25, 50, or 100 μg/mL) for 4 h, and were subsequently exposed to APAP (20 mM) for 24 h. (**A**) The effect of fucoidan on mitochondrial membrane potential was tested using the JC-1 method and determined by a confocal microscope. The J-aggregates produced red fluorescence, and the monomer emitted green fluorescence (Bar = 25 µm). The nuclei were stained with DAPI (blue). (**B**,**C**) Expression of Bax, (**D**–**G**) phosphorylated ASK1, and phosphorylated JNK were determined by Western blotting analysis. Quantification of relative protein expression was performed by densitometric analysis, and α Tubulin was used as an internal control. Similar results were obtained from three independent experiments. All results were expressed as mean ± SD, *n* = 3, *^##^ p* < 0.01 versus control group; * p < 0.05, ** p < 0.01 versus APAP-treated group.

In the case of an APAP overdose, high level of NAPQI depletes cellular glutathione and forms protein adducts, especially on mitochondria, which then inhibits the electron transport chain, resulting in leakage of electrons and oxidative stress [28]. The decline of mitochondrial membrane potential (MMP) is a landmark event in the early stages of mitochondria dysfunction. Compared to the control group, red fluorescence intensity produced by J-aggregates was markedly attenuated after APAP treatment, while incubation with fucoidan alleviated this situation (Figure 7A). As a pro-apoptotic protein, Bax was induced by APAP, and the increased protein expression was inhibited in fucoidan-treated HL-7702 cells (Figure 7B,C). Apoptosis signal-regulating kinase 1 (ASK1) has been identified in a JNK cascade during APAP-induced hepatotoxicity [29]. After APAP incubation, the levels of phosphorylated ASK1 and JNK were also enhanced, and meanwhile being pretreated with fucoidan suppressed phosphorylation of ASK1 and JNK (Figure 7D–G). Taken together, these results indicated that the protective effect of fucoidan against APAP-induced injury might be associated with alleviating mitochondria dysfunction.

### 2.8. Fucoidan Enhanced Nrf2 Expression in the Nucleus of HL-7702 Cells

**Figure 8:**
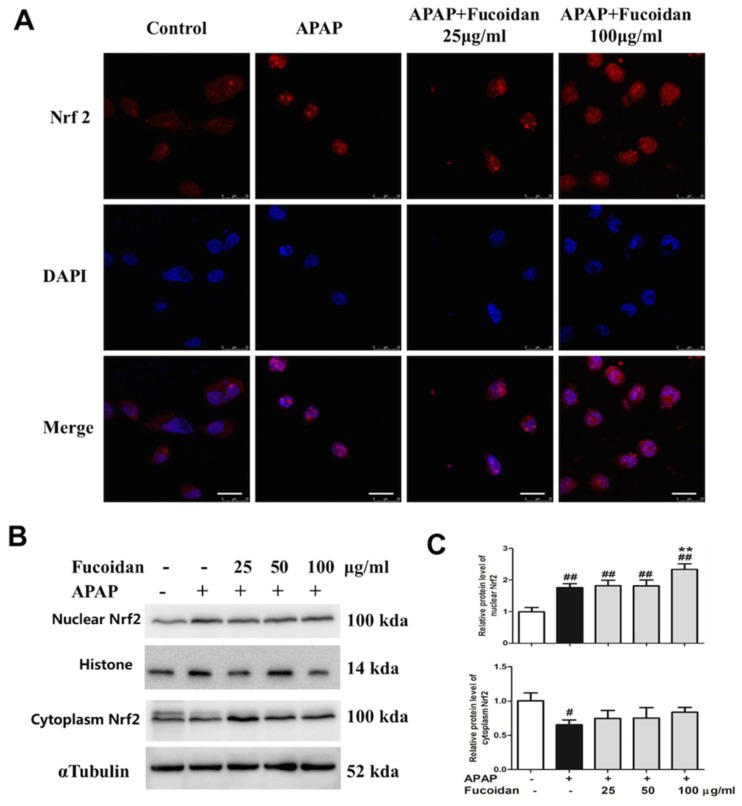
Effect of fucoidan on APAP-induced nuclear factor-erythroid 2-related factor 2 (Nrf2) expression and translocation in HL-7702 cells. Cells were treated with various concentrations of fucoidan (25, 50, or 100 μg/ml) for 4 h, and were subsequently exposed to APAP (20 mM) for 24 h. (**A**) The effect of fucoidan on Nrf2 (red) translocation was tested by immunofluorescence staining and determined by a confocal microscope. The nuclei were stained with DAPI (blue) (Bar = 25 µm). (**B**,**C**) Expression levels of nuclear Nrf2 and cytoplasm Nrf2 were determined by Western blotting analysis. Quantification of relative protein expression was performed by densitometric analysis, with histone and α Tubulin acting as controls. Similar results were obtained from three independent experiments. All results were expressed as mean ± SD, *n* = 3, *^##^ p* < 0.01 versus control group; ** p < 0.01 versus APAP-treated group.

Oxidation damage is one major factor in APAP-induced liver injury. Since the Nrf2-mediated signaling pathway is essential for the inhibition of oxidative stress, Nrf2 plays an important role in the amelioration of APAP-induced hepatotoxicity. Therefore, we examined the expression of Nrf2 in the cytoplasm and nucleus of HL-7702 cells from different groups by immunofluorescence staining and Western blot assay. Exposed to APAP, the expression of Nrf2 in the nuclei remarkably increased, while that in the cytoplasm significantly decreased. In addition, pretreatment with fucoidan further enhanced the expression of Nrf2 in the nucleus, but had no significant effect on Nrf2 in the cytoplasm (Figure 8). In brief, protein expression of total Nrf2 and nuclear Nrf2 were enhanced by fucoidan pretreatment, which might provide a possible mechanism for fucoidan to alleviate APAP-induced oxidative stress and thus protect against APAP hepatotoxicity.

## 3. Discussion 

Currently, APAP overdose-induced hepatotoxicity has become one of the most common causes of acute liver failure in many countries. NAC, the only Food and Drug Administration (FDA)-approved antidote, is used for treatment of APAP hepatotoxicity when administered within 8 to 10 h after APAP overdose [30]. Since the therapeutic options for this disease are rather limited, it is urgent to seek safe and effective agents for the treatment. Fucoidan, which is available for use in cosmetics, functional foods, and dietary supplements, is nontoxic and can be easily extracted from brown seaweeds [31]. A large number of studies have demonstrated that oral or intraperitoneal injection of fucoidan inhibits metastases in various cancers [32]. It has also been reported that fucoidan ameliorated steatohepatitis and insulin resistance by suppressing oxidative stress in experimental non-alcoholic fatty liver disease [33]. In addition, fucoidan also possesses protective effects against CCl_4_-induced liver injury by inhibiting oxidative stress [34]. Due to its broad spectrum of desirable biological functionalities, fucoidan has become one of the extensively studied macromolecules in the last few decades. 

The liver is an important metabolic organ that is able to be impaired or even to be pathologically damaged by various chemical reagents and drugs [35]. Oxidative stress is generally seen in several liver diseases, and ROS plays important roles in the pathogenesis of APAP-induced hepatotoxicity. The prevention of ROS generation and lipid peroxidation is the most common mechanism of hepatoprotective natural compounds [36]. In the current study, mice subjected to fucoidan pretreatment showed significantly decreased lipid peroxidation by scavenging ROS generation. On the other hand, there is an antioxidant system existing to protect the body against oxidative stress. GSH conjugation is the major detoxification pathway for the reactive metabolites generated from APAP. SOD, an important antioxidant enzyme, converts superoxide anion radical induced by NAPQI into hydrogen peroxide (H_2_O_2_) and oxygen. CAT catalyzes the reaction that removes excess H_2_O_2_ in mitochondria. In the present study, GSH level, SOD, and CAT activities in liver tissues were significantly reduced after APAP administration, and meanwhile, treatment with fucoidan significantly alleviated the reduction at 4 h APAP exposure. However, the effect of fucoidan on the enhancement of GSH content was not obvious after 24 h of APAP treatment in vivo (Figure 2). Meanwhile, APAP-induced elevations of serum ALT and AST levels continued until 48 h after APAP administration, whereas the increased serum levels of ALT and AST were significantly inhibited in the mice treated with fucoidan or bicyclol (Figure 1). In addition, pretreatment with fucoidan also significantly alleviated the reduction in GSH level, SOD, and CAT activities at 24 h after APAP exposure in vitro (Figure 5). Thus, the protective effects of fucoidan associated with its antioxidant properties might play a role in the early phase of liver injury, although the effects of fucoidan on APAP-induced hepatotoxicity could last for 48 h or longer (Figure 1). Since NAPQI is mainly metabolized by the CYP pathway, especially CYP2E1, the antioxidant properties of fucoidan in APAP hepatotoxicity might be partially associated with the suppression of CYP enzymes (Figure 6).

JNK, which is expressed in a variety of tissues, is activated in response to a variety of stress stimuli, including DNA damage, growth factors, oxidative, and genotoxic stresses [29]. Additional NAPQI leads to increased ROS accumulation (triggering JNK phosphorylation), further amplifies the mitochondria oxidative stress, and triggers mitochondrial dysfunction [23]. Bax is a pro-apoptotic Bcl-2 family member located predominantly in the cytosol. Exposed to an APAP overdose, it is highly expressed [24,25,26]. It has been reported that APAP-induced mitochondrial Bax expression was attenuated by the inhibition of JNK activation [11]. Moreover, the upstream kinases of JNK activation have been reported to be activated by ASK1 in APAP-induced liver injury [37]. The hepatotoxicity induced by an overdose of APAP was suppressed in ASK knockout mice [38]. Actually, the release of apoptosis inducing factor (AIF) and endonuclease G into the cytosol and the induction of mitochondrial permeability transition were caused by amplified oxidative stress combined with translocation of Bax from the cytosol to the mitochondria [28]. Our data demonstrated that ASK1 and JNK phosphorylation and Bax protein expression were markedly inhibited in fucoidan-pretreated groups after APAP administration (Figure 3 and Figure 7), which suggests that fucoidan reducing APAP-induced toxicity might be associated with alleviating mitochondria dysfunction. 

**Figure 9:**
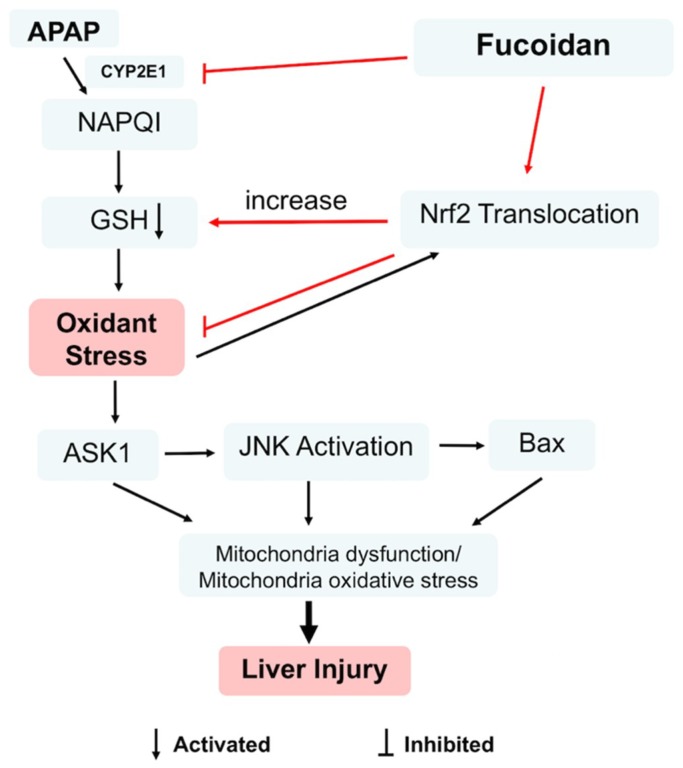
Nrf2 was involved in the protective effect of fucoidan against acetaminophen-induced hepatotoxicity. Fucoidan could regulate Nrf2 translocation, which contributes to the inhibition of APAP-induced oxidant stress. In addition, the inhibition of CYP2E1 by fucoidan might be related to the enhancement of GSH levels. Moreover, fucoidan attenuated the phosphorylation of ASK1 and JNK and further decreased the protein expression of Bax. Subsequently, fucoidan alleviated mitochondria dysfunction and protected hepatocytes against APAP toxicity.

Besides SOD, GSH, and CAT, Nrf2 also belongs to the antioxidant system, which maintains the balance of ROS in hepatocytes. As a key nuclear transcription factor, Nrf2 enters the nucleus from the cytoplasm when exposed to stressors or inducers. Subsequently, Nrf2 binds to ARE and regulates the expression of a battery of cytoprotective genes encoding intracellular detoxifying enzymes, including SOD, GSH, and CAT, which are responsible for APAP elimination and detoxification [39,40]. In the current study, APAP treatment increased the translocation of Nrf2. However, pretreatment with fucoidan further upregulated nuclear Nrf2 in HL-7702 cells (Figure 8). These observations revealed that fucoidan pretreatment efficiently stimulated Nrf2 translocation from the cytoplasm into the nucleus, then enhanced the ability of antioxidant stress and suppressed APAP-induced ROS accumulation (Figure 9). 

In summary, the present study provided an investigation into the protective activities of fucoidan against APAP-induced liver injury, and the potential mechanism was upregulating the Nrf2 antioxidant pathway. Thus, our study suggested a possible therapeutic application of fucoidan in APAP hepatotoxicity.

## 4. Materials and Methods 

### 4.1. Chemicals and Reagents 

Fucoidan (purity >98%) was obtained from Cool Chemistry CO., Ltd (Beijing, China). APAP was provided by Santa Cruz Biotechnology (Santa Cruz, CA, USA). Bicyclol was obtained from Beijing Union Pharmaceutical Plant (Beijing, China). ALT, AST, MDA, GSH, CAT, and SOD activity test kits were from Nanjing Jiancheng Bioengineering Institute (Nanjing, China). ROS Fluorescent Probe-Dihydroethidium (DHE) was from Vigorousbio. CO., Ltd. (Beijing, China). The mitochondrial membrane potential assay kit (JC-1 Kit) and the LDH kit were obtained from Beyotime Biotechnology (Haimen, China). Antibodies against Nrf2, CYP 2E1, and ASK1 were obtained from Abcam (Cambridge, United Kingdom). Antibodies against histone, pASK1, pJNK, JNK, and Bax were obtained from Cell Signaling Technology (Beverly, MA, USA). Antibody against α tubulin was from Proteintech (Wuhan, China).

### 4.2. Animals and Treatment

ICR mice were obtained from Nantong University Experimental Animal Center. Animal experiments were performed in accordance with the National Institutes of Health (NIH) guidelines for Care and Use of Laboratory Animals. The study was approved by the University Animal Ethics Committee of Nantong University (approval no. NTU-20170316, 16 March 2017) and was conducted in accordance with the Declaration of Helsinki. 

After 1 week of adaptive rearing, 6-week-old male ICR mice were divided into control, model, fucoidan (100 and 200 mg/kg), and bicyclol 200 mg/kg groups. The drugs were dissolved in saline and orally administered for 7 consecutive days. The mice in the control and model groups were intragastrically administered with an equivalent volume of saline. Two hours after final administration of medication, the mice were intraperitoneally injected with 500 mg/kg APAP [41], and the normal group was injected with an equal amount of saline. The mice continued to be administered fucoidan or bicyclol once per day, and were sacrificed at an indicated time. 

### 4.3. Cell Culture and Treatment

A human normal hepatocyte HL-7702 cell line was obtained from the China Cell Line Bank (Beijing, China). The HL-7702 cells were cultured in Dulbecco modified Eagle medium (DMEM) medium containing 10% fetal bovine serum (FBS), 100 U/mL of streptomycin, 100 U/mL of penicillin, and 3 mM glutamine. The cells were grown in a humidified atmosphere containing 5% CO_2_ at 37 °C. Besides the control and APAP group, all the cells were treated with fucoidan (25, 50, and 100 μg/mL) for 4 h and were exposed to APAP (20 mM) for 24 h. 

### 4.4. Biochemical Indexes Assay 

All mice were killed 4 h after APAP injection, and the serum and liver were collected for detection of biochemical indexes. For HL-7702 cells, medium and cells were collected at 24 h after APAP administration. The levels of ALT and AST were determined according to the manufacturer’s protocol available with the respective kits. In addition, live tissues and cells were homogenized to analyze the MDA, SOD, CAT, and GSH levels in accordance with the manufacturer’s instructions. All the results were normalized by the total protein concentration in each sample.

### 4.5. MTT and LDH Analysis

Cell viability was determined by MTT assay according to the manufacturer’s instructions. Briefly, the HL-7702 cells were plated into 96-well plates and treated with APAP for 24 h. After the incubated period, MTT (5 mg/mL) was added to each well and incubated for another 4 h. Then the supernatant was removed, and dimethyl sulphoxide (DMSO) was used to lyse the cells. The absorbance values of each group were measured at 570 nm. LDH levels were detected with an LDH Cytotoxicity Assay Kit, following the manufacturer’s protocol. The absorbance was measured at 490 nm.

### 4.6. Histology and Immunofluorescence

The livers were harvested, fixed in 4% paraformaldehyde, and embedded in paraffin. Hematoxylin and eosin (H&E) staining was used to detect the degree of liver injury. For immunofluorescence staining, cells were fixed with 4% paraformaldehyde and washed with 0.01 M PBS three times, then treated with PBS containing 0.3% Triton-X-100 for 30 min. After blocking with 4% bovine serum albumin (BSA) for 1 h, an antibody against Nrf2 (1:100) was added and incubated overnight at 4 °C. The next day, cells were washed three times with 0.01 M PBS and incubated with Alexa Fluor 488-conjugated immunoglobulin G (IgG) (1:1000) for 2 h at room temperature. The nuclei were stained with DAPI. The images were finally captured with a laser confocal fluorescence microscope. 

### 4.7. DHE Staining

Liver tissue was embedded in an optimal cutting temperature compound, performed on a frozen section. Approximately 6 μm thick sections were washed with 0.01 M PBS twice. DHE (2 μM/L) in hydroxyethyl piperazine ethanesulfonic acid (HEPES) buffer was added on the tissue and mixed well, and placed in the dark at 37 °C for 30 min, then washed with PBS three times. For HL-7702 cells, DHE was directly added into the medium, and the final concentration was 2 μM/L. After incubation in the dark for 30 min, the cells were washed with PBS. Laser confocal fluorescence microscopy was used to photograph the fluorescence intensity.

### 4.8. JC-1 Assay for Mitochondrial Membrane Potential (MMP)

MMP was measured by JC-1 staining. HL-7702 cells were seeded into 12-well plates at a density of 2 × 10^5^ cells/well for 12 h. Subsequently, the cells were subjected to different dosages of fucoidan (25 or 100 μg/mL) for 4 h, followed by exposure to 20 mM APAP for 24 h. Next, the cells were washed with PBS and incubated with JC-1 (10 μg/mL) at 37 °C in the dark for 20 min. Photos were taken by a laser confocal fluorescence microscope.

### 4.9. Western Blot Analysis

Hepatic tissues and cells were homogenized and lysed in Radio Immunoprecipitation Assay (RIPA) Lysis buffer supplemented with phenylmethanesulfonyl fluoride (1:100) for 30 min on ice, and the protein concentration was determined using a bicinchoninic acid (BCA) kit (Haimen, China). Cytosolic and nuclear extracts were prepared with a commercially available kit (Beyotime Biotechnology, Haimen, China) in accordance with the manufacturer’s instructions. Equal amounts of extracted protein (10–40 μg) were separated on 10% or 12% sodium dodecyl sulfate polyacrylamide gel electrophoresis (SDS-PAGE), and proteins were transferred to a polyvinylidene fluoride (PVDF) membrane and blocked with tris-buffered saline and Tween 20 (TBST) containing 5% nonfat milk for 2 h at room temperature. Subsequently, the membranes were incubated overnight at 4 °C with primary antibodies and incubated with secondary antibodies for 2 h at room temperature. The relative protein levels were calculated by quantification of band intensity with a Bio-red imaging system (Bio-red, Berkeley, CA, USA). 

### 4.10. Statistical Analysis 

Data are represented as mean ± SD. Statistical differences were assessed by one-way ANOVA analysis of Tukey’s multiple comparison test using GraphPad Prism 7.0 software (San Giego, CA, USA), and *p* < 0.05 was considered statistically significant.

## Abbreviations

APAP	acetaminophen
ALT	alanine aminotransferase
AST	aspartate aminotransferase
ROS	reactive oxygen species
MDA	malondialdehyde
SOD	superoxide dismutase
GSH	glutathione
CAT	catalase
MMP	mitochondrial membrane potential
NAQPI	*N*-acetyl-q-benzoquinoneimine
JNK	c-Jun N-terminal protein kinases
Nrf2	nuclear factor-erythroid 2-related factor 2
NAC	*N*-acetylcysteine
ASK1	apoptosis signal-regulating kinase 1
DHE	dihydroethidium
